# Prognostic and clinicopathological roles of programmed death‐ligand 1 (PD‐L1) expression in thymic epithelial tumors: A meta‐analysis

**DOI:** 10.1111/1759-7714.13590

**Published:** 2020-09-14

**Authors:** Hyun Min Koh, Bo Gun Jang, Hyun Ju Lee, Chang Lim Hyun

**Affiliations:** ^1^ Department of Pathology Gyeongsang National University Changwon Hospital Changwon Korea; ^2^ Department of Pathology Jeju National University School of Medicine Jeju Korea; ^3^ Department of Pathology Jeju National University Hospital Jeju Korea; ^4^ Department of Pathology Soonchunhyang University College of Medicine Cheonan Korea; ^5^ Department of Pathology Soonchunhyang University Cheonan Hospital Cheonan Korea

**Keywords:** Meta‐analysis, prognosis, programmed death‐ligand 1, thymic epithelial tumor

## Abstract

**Background:**

Programmed death‐ligand 1 (PD‐L1) is one of the immune checkpoint proteins, and plays an important role in the progression and microenvironment of cancer. PD‐L1 expression has been associated with poor survival in many cancers. Several studies have also shown an association between PD‐L1 expression and the prognosis of patients with thymic epithelial tumors (TETs). In this study, we systematically evaluated the prognostic and clinicopathological roles of PD‐L1 expression in TETs.

**Methods:**

We searched the literature through PubMed, Embase and Cochrane library and chose the eligible studies, and subsequently performed a meta‐analysis to evaluate the prognostic and clinicopathological roles of PD‐L1 expression in TETs.

**Results:**

Six of the 75 articles found in the literature were selected. PD‐L1 expression was significantly related to unfavorable overall survival (hazard ratio 1.52, 95% confidence interval [CI]: 1.01–2.30, *P* = 0.046) in TETs. PD‐L1 expression was significantly associated with male gender (odds ratio [OR] 1.55, 95% CI: 1.08–2.22, *P* = 0.017) and higher Masaoka stage (OR 3.93, 95% CI: 2.44–6.32, *P* < 0.001).

**Conclusions:**

PD‐L1 expression was correlated with unfavorable prognosis in TETs, indicating PD‐L1 expression could help determine the prognosis of TET patients.

## Introduction

Thymic epithelial tumors (TETs), including thymoma and thymic carcinoma (TC), are rare malignant tumors that show various morphologic appearances and clinical symptoms.^1^ They are the most common mediastinal tumors in adult, with a reported incidence of 0.05 per 100 000 person‐years.^2^ Complete resection is the first choice of treatment for TETs; however approximately 10%–30% of patients with TETs experience recurrence after surgery.^1^, ^3^ Various treatments have been tried in patients where surgery is not an option or in whom there has been disease recurrence, but the clinical outcomes are inconclusive.^3^

Programmed death‐ligand 1 (PD‐L1) is one of the immune checkpoint proteins, and plays important roles in the progression and microenvironment of the cancer.^4^ Clinical trials have shown favorable results of PD‐L1 targeting immunotherapy in some malignancies.^5^ Recently, several studies of anti‐PD‐L1 therapy for TETs have found that about a quarter of the patients had a good response.^1^

PD‐L1 expression has been associated with poor survival in many cancers, including lung, pancreatic, esophageal, breast, ovarian, bladder, renal cancers and hematologic malignancies.^6^ Several studies have also shown an association between PD‐L1 expression and prognosis of patients diagnosed with TETs.^1^, ^3^, ^4^, ^7^, ^8^, ^9^ However, the association of PD‐L1 expression with the prognosis of TETs has not yet been systematically analyzed.

Therefore, we performed a comprehensive meta‐analysis to explore the prognostic and clinicopathological roles of PD‐L1 expression in TETs.

## Methods

### Search strategy

We searched the literature through PubMed, Embase and Cochrane library and chose eligible studies to include in the analysis. The search was conducted until 1 May 2020 using the following keywords: PD‐L1 or programmed death‐ligand 1; and thymic carcinoma or thymic epithelial tumor; and prognostic or predict or prognosis; or survival or outcome. This was accompanied by a manual search.

### Inclusion and exclusion criteria

The analysis included studies satisfying the following conditions: (i) A study showing the correlation of PD‐L1 expression with prognosis in human TETs; and (ii) hazard ratio (HR) with 95% confidence interval (CI) was reported. Articles with inaccurate data, together with conference abstracts and reviews were excluded from the analysis.

### Data extraction and quality assessment

We collected basic information reviewing the included articles. Two authors collected basic information individually and reached an agreement if there were any differences in the results. We evaluated the quality of the included articles using the Newcastle‐Ottawa Scale (NOS). The quality assessment was also conducted separately by two authors, and if there were any differences in the results, an agreement was reached.

### Statistical analysis

We conducted a meta‐analysis using StataSE12 (Stata, College Station, TX, USA). I^2^ value was used to determine the degree of heterogeneity among the included studies. We also performed a funnel plot with filled method and Egger test to reveal the publication bias. Sensitivity analysis was performed to confirm the consistency of the pooled results. A *P*‐value <0.05 was considered as statistically significant.

## Results

### Basic information of the included studies

**Figure 1:**
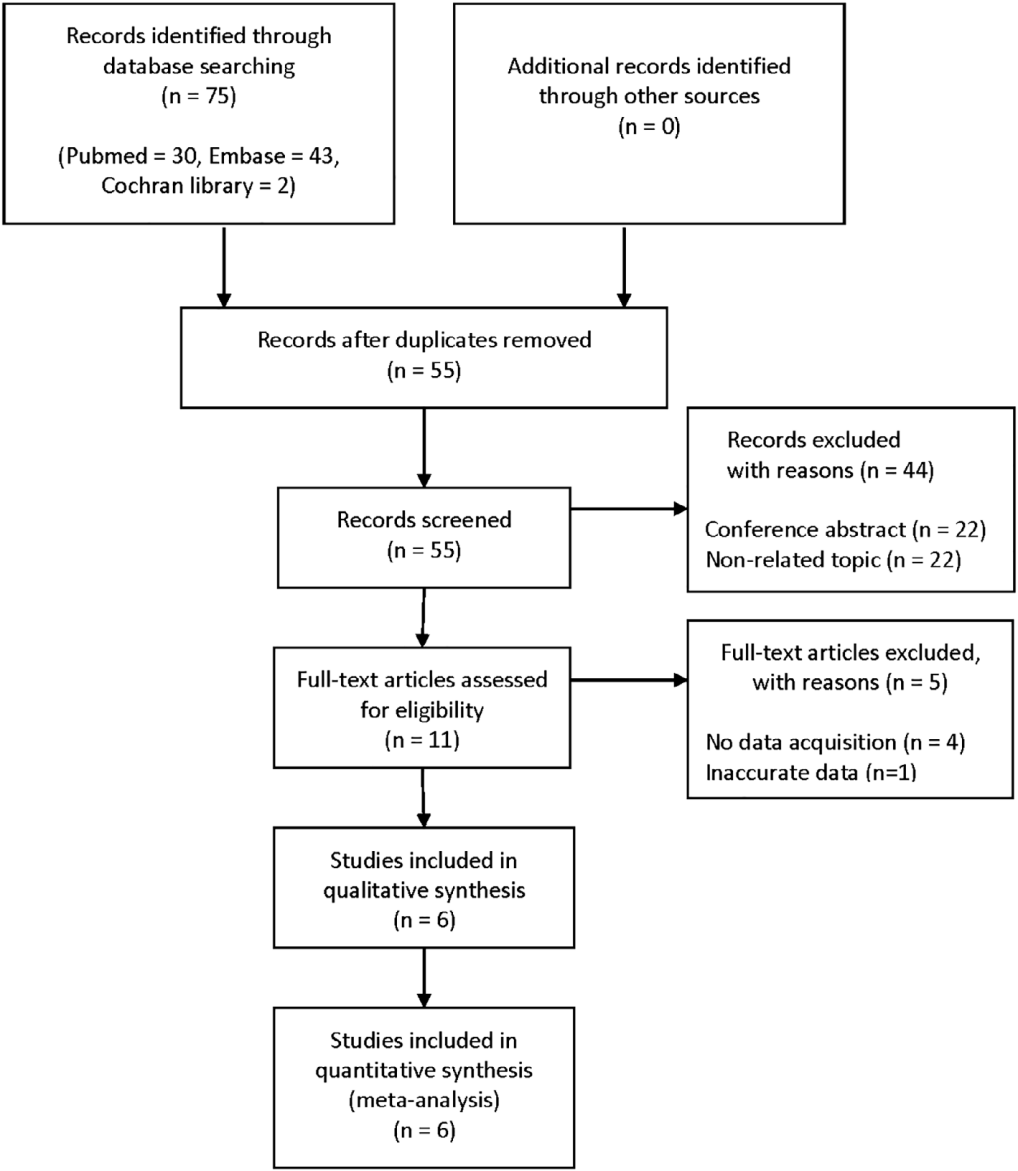
Flow diagram of study selection.

Six studies of the 75 articles found in the literature were selected (Fig 1) The basic information of included studies is given in Table 1 and a total of 772 patients with TETs were included. One study included TC, two studies included thymoma, and the other three included both tumors. In all studies, PD‐L1 expression was confirmed through immunohistochemical staining. The quality score of the included studies was 7 to 8 points.

**Table 1:** Basic information of the included studies

Study	Country	Tumor type	Sample size	Gender (Male/female)	Mean or median age (years)	Masaoka stage	Treatment	Study period	Mean or median follow‐up (months)	Survival outcome	PD‐L1 detection method	Cutoff value of PD‐L1 expression	Survival analysis	NOS
Funaki *et al*. (2019)^4^	Japan	TC	43	26/10	NA	I–IV	Surgery and/or induction chemotherapy and/or radiation	1996–2017	51	DFS	IHC	≥50%	UVA	7
Hakiri *et al*. (2019)^7^	Japan	Thymoma	81	41/40	61 (25–81)	I–IV	Surgery and/or induction chemotherapy	2004–2015	37 (1–137)	OS, DFS	IHC	>1%	MVA	8
Song *et al*. (2019)^1^	South Korea	TET	302 (thymoma) 60 (TC)	193/169	52 (thymoma) 54 (TC)	I–IV	Surgery and neoadjuvant and/or postoperative adjuvant radiation and chemotherapy	1996–2014	NA	OS (thymoma)	IHC	≥50%	MVA	7
Owen *et al*. (2018)^8^	USA	TET	32 (thymoma) 3 (TC)	18/17	55 (33–71)	I–IV	Surgery	NA	74	OS (TC)	IHC	≥3 (semi‐quantitative scoring system)	UVA	7
Wei *et al*. (2018)^3^	Taiwan	TET	100 (thymoma) 69 (TC)	62/38	52.5 (thymoma) 55 (TC)	I–IV	Surgery and neoadjuvant and/or postoperative adjuvant radiation and/or chemotherapy	1988–2013	78.7 (thymoma) 43 (TC)	OS, PFS (thymoma, TC)	IHC	3+ 2+ and >50%	MVA	8
Yokoyama *et al*. (2016)^9^	Japan	Thymoma	82	32/50	60.5 (27–82)	I–IV	Surgery and/or radiation and/or chemotherapy	2000–2013	34 (1–144)	DFS	IHC	>38%	MVA	8

DFS, disease‐free survival; IHC, immunohistochemistry; MVA, multivariate analysis; NA, not available; NOS, Newcastle‐Ottawa Scale; OS, overall survival; PD‐L1, programmed death‐ligand 1; PFS, progression‐free survival; TC, thymic carcinoma; TET, thymic epithelial tumor; UVA, univariate analysis.

### Association between PD‐L1 expression and overall survival

The analysis of the association between PD‐L1 expression and overall survival (OS) included four studies with 555 TET patients. Hakiri *et al*.^7^ and Song *et al*.^1^ reported the HR in thymoma, Owen *et al*.^8^ in TC, and Wei *et al*.^3^ in both. This analysis was performed to include each HR reported in Wei *et al*.^3^

**Figure 2:**
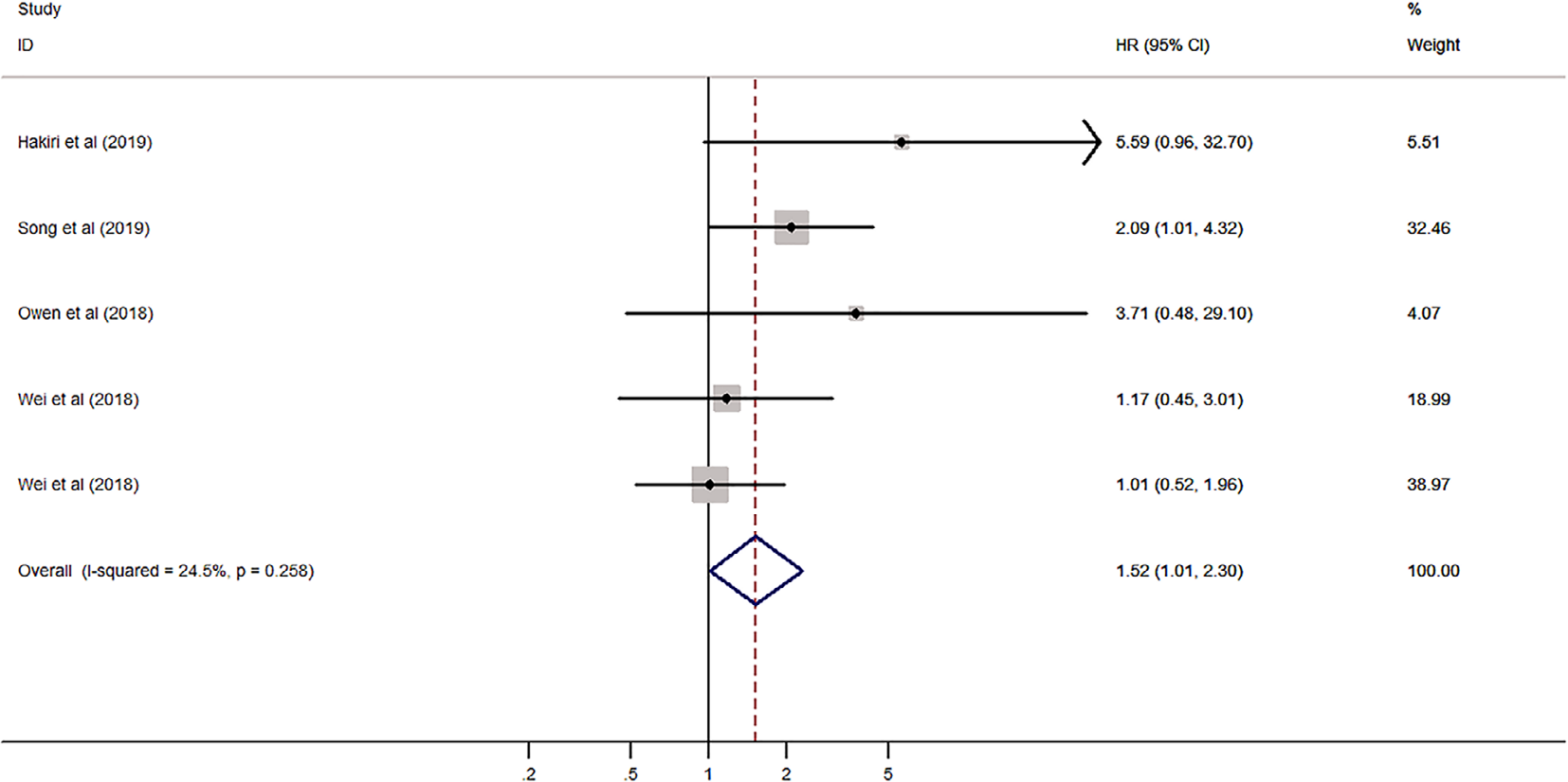
Forest plot of the association between PD‐L1 expression and overall survival (OS).

**Figure 3:**
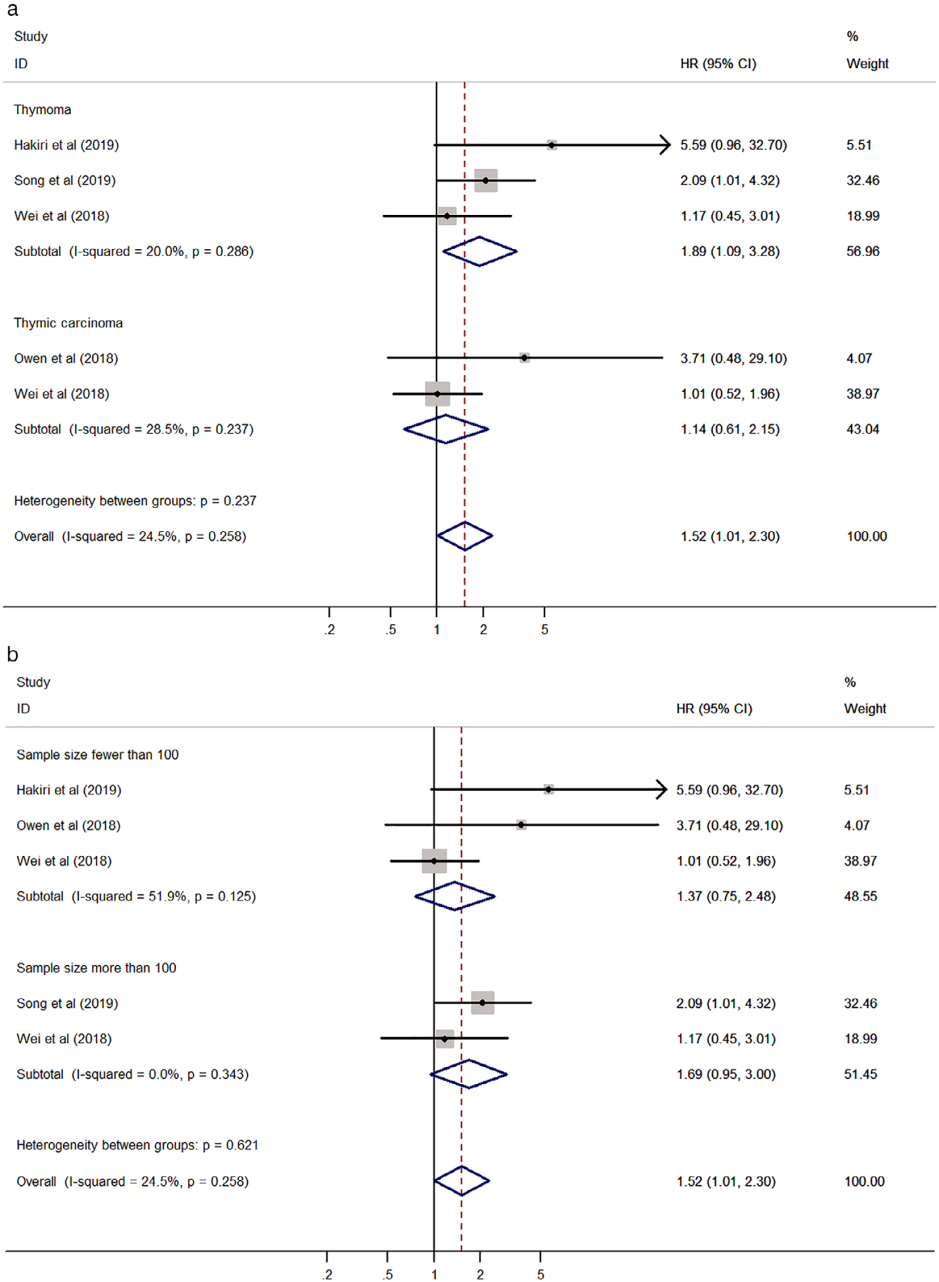
Forest plot of the association between PD‐L1 expression and overall survival (OS) stratified by (**a**) tumor type; and (**b**) sample size.

The pooled HR was evaluated using a fixed effect model (I^2^ = 24.5%, *P* = 0.258). The pooled HR was 1.52 (95% CI: 1.01–2.30, *P* = 0.046), implying there was a relationship between PD‐L1 expression and unfavorable OS in TETs (Fig 2). We also identified that PD‐L1 expression could be an independent prognostic factor for OS in thymoma patients through multivariate analysis (HR 1.89, 95% CI: 1.09–3.28, *P* = 0.023). Additionally, we conducted subgroup analyses according to tumor type (thymoma vs. TC) and sample size (fewer than 100 vs. more than 100). The results showed that the relationship between PD‐L1 expression and poor OS was still significant in the group with thymoma (HR 1.89, 95% CI: 1.09–3.28, *P* = 0.023) (Table 2) (Fig 3a). No significant results were obtained in the groups with TC and sample size (Table 2) (Fig 3a,b).

**Table 2:** Subgroup analysis of the association between PD‐L1 expression and overall survival (OS) in thymic epithelial tumors (TETs)

					Heterogeneity	
Subgroup	Number of studies	Number of patients	Pooled HR (95% CI)	*P*‐value	I^2^ (%)	*P*‐value
Tumor type						
Thymoma	3	483	1.89 (1.09–3.28)	0.023	20.0	0.286
Thymic carcinoma	2	72	1.14 (0.61–2.15)	0.680	28.5	0.237
Sample size						
Fewer than 100	3	153	1.37 (0.75–2.48)	0.302	51.9	0.125
More than 100	2	402	1.69 (0.95–3.00)	0.076	0.0	0.343

CI, confidence interval; HR, hazard ratio; PD‐L1, programmed death‐ligand 1.

### Association between PD‐L1 expression and disease‐free survival

The analysis of the association between PD‐L1 expression and disease‐free survival (DFS) or progression‐free survival (PFS) included four studies with 375 TET patients. Hakiri *et al*.^7^ and Yokoyama *et al*.^9^ reported the HR in thymoma, Funaki *et al*.^4^ in TC, and Wei *et al*.^3^ in both. In this meta‐analysis, PFS was regarded as DFS, and the HRs reported by Wei *et al*.^3^ in both types of tumors was included.

**Figure 4:**
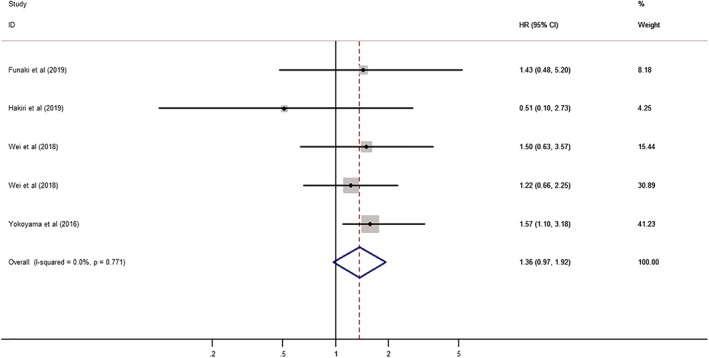
Forest plot of the association between PD‐L1 expression and disease‐free survival (DFS).

**Figure 5:**
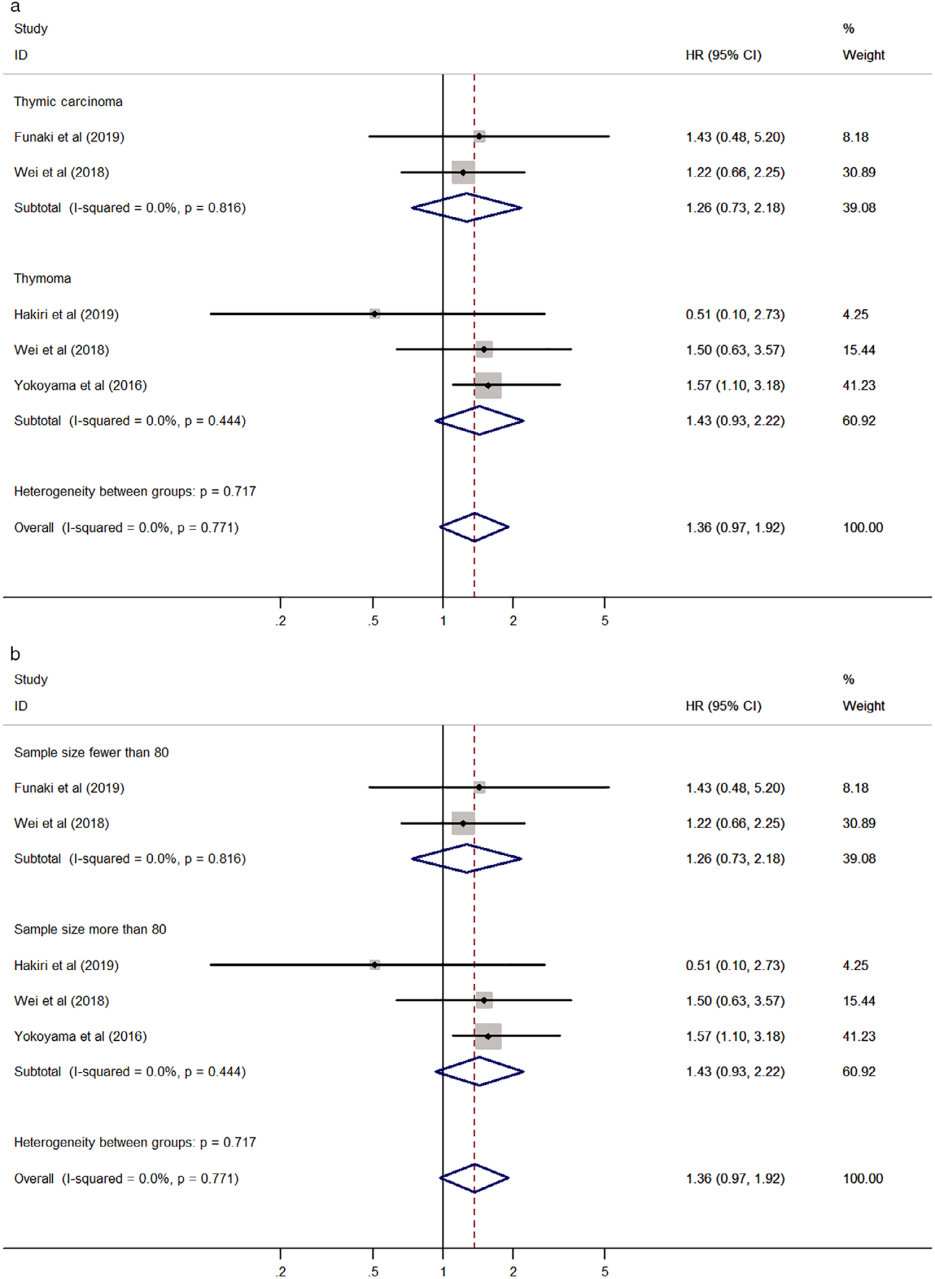
Forest plot of the association between PD‐L1 expression and disease‐free survival (DFS) stratified by (**a**) tumor type; and (**b**) sample size.

The association between PD‐L1 expression and DFS was analyzed as a fixed effect model because of the low heterogeneity between the included studies (I^2^ = 0.0%, *P* = 0.771). The pooled HR was 1.36 (95% CI: 0.97–1.92, *P* = 0.074) (Fig 4). In subgroup analyses according to tumor type (thymoma vs. TC) and sample size (fewer than 80 vs. more than 80), no groups showed significant results (Table 3) (Fig 5a,b).

**Table 3:** Subgroup analysis of the association between PD‐L1 expression and disease‐free survival (DFS) in thymic epithelial tumors (TETs)

					Heterogeneity	
Subgroup	Number of studies	Number of patients	Pooled HR (95% CI)	*P*‐value	I^2^ (%)	*P*‐value
Tumor type						
Thymoma	3	263	1.43 (0.93–2.22)	0.105	0.0	0.444
Thymic carcinoma	2	112	1.26 (0.73–2.18)	0.404	0.0	0.816
Sample size						
Fewer than 80	2	112	1.26 (0.73–2.18)	0.404	0.0	0.816
More than 80	3	263	1.43 (0.93–2.22)	0.105	0.0	0.444

CI, confidence interval; HR, hazard ratio; PD‐L1, programmed death‐ligand 1.

### Association between PD‐L1 expression and clinicopathological factors

**Figure 6:**
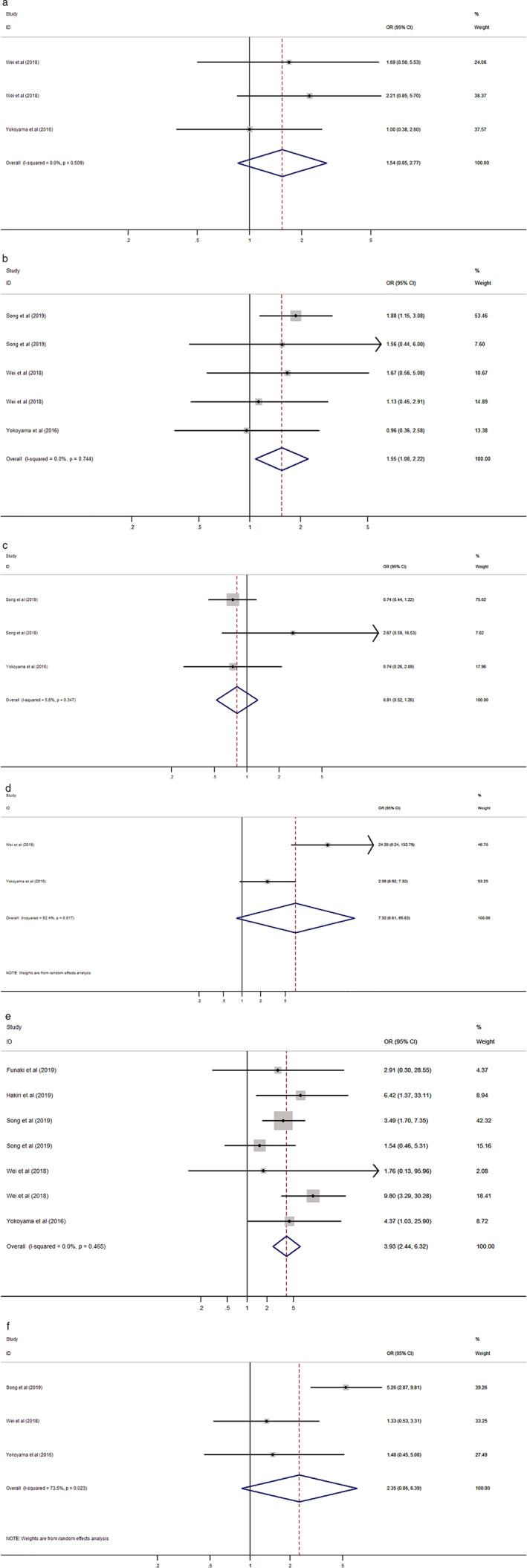
Forest plot of the association between PD‐L1 expression and clinicopathological factors. (**a**) Age; (**b**) gender; (**c**) tumor size; (**d**) tumor grade; (**e**) Masaoka stage; and (**f**) myasthenia gravis.

PD‐L1 expression was significantly associated with male gender (odds ratio [OR] 1.55, 95% CI: 1.08–2.22, *P* = 0.017) and higher Masaoka stage (OR 3.93, 95% CI: 2.44–6.32, *P* < 0.001), but not with age, tumor size and grade, and myasthenia gravis (Table 4, Fig 6a–f).

**Table 4:** Association between PD‐L1 expression and clinicopathological factors in patients with thymic epithelial tumors (TETs)

Factor	Number of studies	Number of patients	Pooled OR (95% CI)	*P*‐value	Heterogeneity		
					I^2^ (%)	*P*‐value	Model
Age (old vs. young)	2	251	1.54 (0.85–2.77)	0.152	0.0	0.509	Fixed
Gender (male vs. female)	3	613	1.55 (1.08–2.22)	0.017	0.0	0.744	Fixed
Tumor size (large vs. small)	2	444	0.81 (0.52–1.26)	0.349	5.6	0.347	Fixed
Tumor grade^*^ (high vs. low)	2	182	7.32 (0.81–65.82)	0.076	82.4	0.017	Random
Masaoka stage (III, IV vs. I, II)	5	737	3.93 (2.44–6.32)	< 0.001	0.0	0.465	Fixed
Myasthenia gravis^*^ (present vs. absent)	3	484	2.35 (0.86–6.39)	0.094	73.5	0.023	Random

CI, confidence interval; OR, odds ratio; PD‐L1, programmed death‐ligand 1.

^*^ The analysis included only thymoma.

### Publication bias

**Figure 7:**
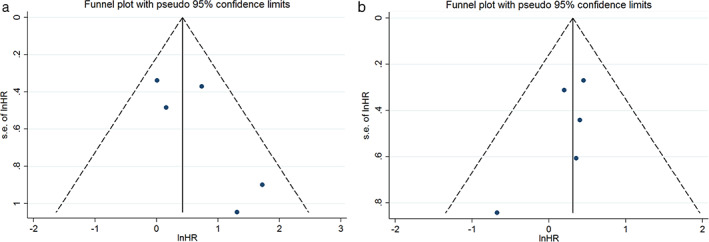
Funnel plot of the association between PD‐L1 expression with (**a**) overall survival (OS); and (**b**) disease‐free survival (DFS).

**Figure 8:**
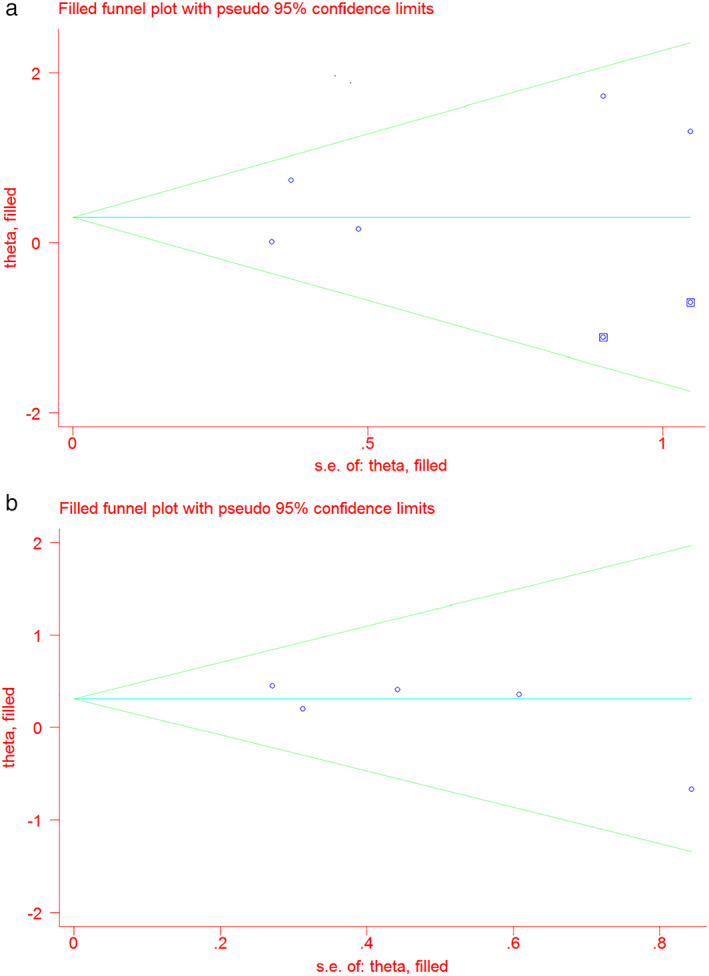
Trim and fill funnel plot of the association between PD‐L1 expression with (**a**) overall survival (OS); and (**b**) disease‐free survival (DFS).

The funnel plots suggested a publication bias, but it was not statistically proven for OS (*P* = 0.184); and for DFS (*P* = 0.234) (Fig 7a,b). Thus, a trim‐and‐fill test was conducted. The pooled HR was 1.35 (95% CI: 0.91–2.01, *P* = 0.153) with two studies filled in for OS, and the result was unchanged for DFS (HR 1.36, 95% CI: 0.97–1.92, *P* = 0.074) (Fig 8a,b).

### Sensitivity analysis

**Figure 9:**
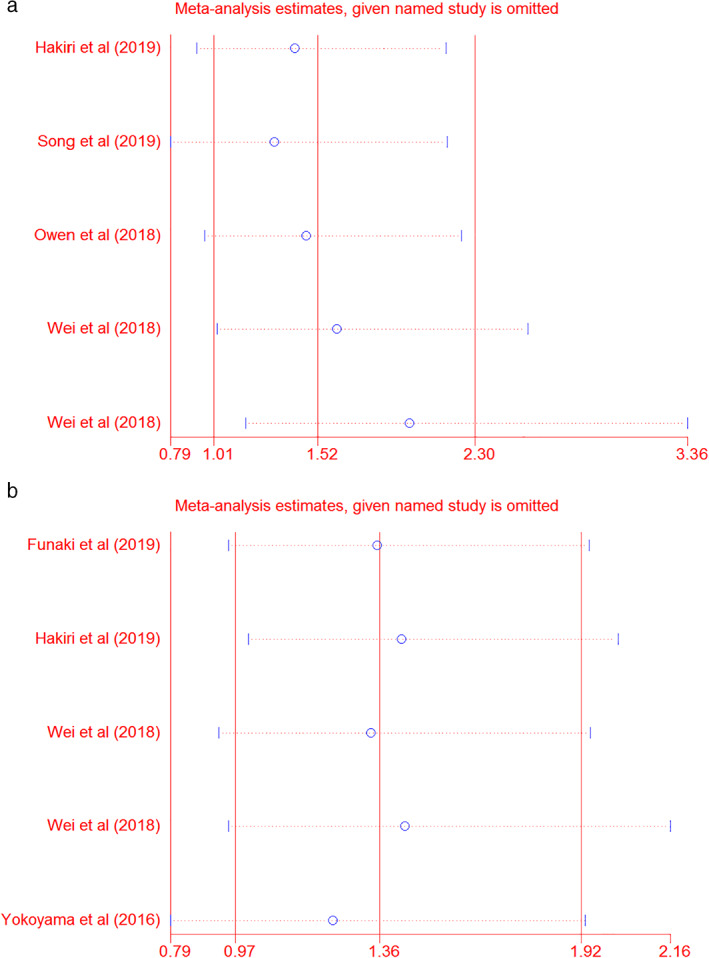
Sensitivity analysis of the association between PD‐L1 expression with (**a**) overall survival; (OS) (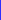) Lower CI limit (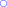) Estimate (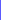) Upper CI limit and (**b**) disease‐free survival (DFS). (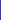) Lower CI limit (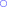) Estimate (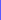) Upper CI limit .

In the sensitivity analyses, the study by Wei *et al*.^3^ in TC showed a major effect of individual study for OS (HR 1.62, 95% CI: 1.02–2.57), and the study by Yokoyama *et al*.^9^ revealed a great impact for DFS (HR 1.24, 95% CI: 0.79–1.93). Nevertheless, the sensitivity analysis proved that our pooled results had not changed at all, suggesting that our results were reliable and consistent for OS (HR 1.52, 95% CI: 1.01–2.30); and for DFS (HR 1.36, 95% CI: 0.97–1.92) (Fig 9a,b).

## Discussion

PD‐L1 is a T cell coinhibitory receptor with a unique biologic function.^10^ PD‐L1 negatively regulates T cell‐mediated immune responses and PD‐L1 activation allows cancer cells to escape from the immune system.^11^ Thus, blocking PD‐L1 makes effective immunotherapy possible.^11^

PD‐L1 is selectively expressed on various cancer and inflammatory cells within the tumor microenvironment.^10^ PD‐L1 expression has been demonstrated in many cancers such as breast and ovarian cancers, pancreatic and esophagus adenocarcinoma, kidney and bladder cancers, lung cancers, melanoma, as well as hematologic malignancies, with evidence of associations with clinicopathological factors and prognosis.^6^, ^12^ Recently, several studies have been reported on the relationship between PD‐L1 expression and prognosis in TETs.

Here, a meta‐analysis was performed for a systematic understanding of the relationship between PD‐L1 expression and prognosis in TETs.

In the analysis of the association between PD‐L1 expression and OS, the results revealed a close correlation between PD‐L1 expression and OS (HR 1.52, 95% CI: 1.01–2.30, *P* = 0.046), suggesting an association between PD‐L1 expression with unfavorable OS in TETs. Nevertheless, in the subgroup analysis, the results were only statistically significant for thymoma (HR 1.89, 95% CI: 1.09–3.28, *P* = 0.023), and not for TC.

With respect to the association between PD‐L1 expression and DFS, the results of the analysis suggested a link between PD‐L1 expression and DFS, but were not statistically significant (HR 1.36, 95% CI: 0.97–1.92, *P* = 0.074).

In addition, PD‐L1 expression was significantly related to male gender (OR 1.55, 95% CI: 1.08–2.22, *P* = 0.017) and higher Masaoka stage (OR 3.93, 95% CI: 2.44–6.32, *P* < 0.001).

Despite various efforts, this study had some limitations. First, there were not many studies related to PD‐L1 expression and prognosis, so the number of studies included in our analysis was small. In particular, the fact that it contains very few cases of TC and the failure to fully consider that TC is a separate disease in terms of natural prognosis and treatment especially chemotherapy is considered a major limitation of our research. Second, all the studies included, except one, were published in Asia, which was also a limitation of our study. Finally, the cutoff value of PD‐L1 expression varied slightly from study to study, so this might have affected our results. Since there is no gold standard for cutoff value of PD‐L1 expression so far and each PD‐L1 assay applies a different automated staining system, detection system, and cutoff value to determine PD‐L1 expression, it seems reasonable that studies with various cutoff values of PD‐L1 expression are reported.^13^ However, we hope to establish common standards through harmonized studies.

In conclusion, PD‐L1 expression was related to poor prognosis and higher stage in TETs. The results indicated that PD‐L1 expression could help determine the prognosis of TET patients.

## Disclosure

No authors report any conflict of interest.
